# Interactions of impulsivity, general executive functions, and specific inhibitory control explain symptoms of social-networks-use disorder: An experimental study

**DOI:** 10.1038/s41598-020-60819-4

**Published:** 2020-03-02

**Authors:** Elisa Wegmann, Silke M. Müller, Ofir Turel, Matthias Brand

**Affiliations:** 1 General Psychology: Cognition and Center for Behavioral Addiction Research (CeBAR), University of Duisburg-Essen, Duisburg, Germany; 2 Erwin L. Hahn Institute for Magnetic Resonance Imaging, Essen, Germany; 3 Information Systems and Decision Sciences, California State University, Fullerton, USA; 4 Brain and Creativity Institute, University of Southern California, Los Angeles, USA

**Keywords:** Human behaviour, Risk factors

## Abstract

While the use of social media and online-communication applications has become an integral part of everyday life, some individuals suffer from an excessive, uncontrolled use of social media despite experiencing negative consequences. In accordance with neuropsychological models of addiction, we assume the tendency of a social-networks-use disorder to be related to an interplay of predisposing personality traits (e.g., impulsivity), and reductions in cognitive functions (e.g., executive functions, inhibitory control). The current study makes first strides towards examining this interplay. In addition to a newly developed social-networks-specific auditory Go-NoGo paradigm, other neuropsychological paradigms were used. Impulsivity and social-networks-use-disorder symptoms were assessed by standardized questionnaires. The results show that the symptom severity of a social-networks-use disorder is mainly associated with attentional impulsivity. General executive functions and specific inhibitory control of social-networks-related cues have no direct effect on symptom severity. However, moderated regression analyses emphasize that increased symptom severity is associated with higher attentional impulsivity, especially if there are additionally reductions in executive functions or specific inhibitory control. The results complement previous findings and inform future research on social-networks-use disorder. The findings support the applicability of theoretical models of addictive behaviors to the social-networks-use disorder and point to social-networks-related specificities regarding attention-related facets.

## Introduction

In 2016, there were 2.16 billion smartphone users worldwide and experts expect the number to rise to over 3.01 billion users by the year 2021^1^. Such devices typically generate auditory cues (ringtones) to inform people about new messages; and these are generated many times per day, anytime and anywhere^2^. Such ringtones indicate incoming calls, messages, or other novel information provided by online applications; and are associated with an implicit or explicit demand of a specific action, i.e., interacting with the smartphone^3^. One of the most popular applications used on smartphones are social-media applications or online-communication applications (e.g., WhatsApp, Instagram, Twitter)^4^, which allow users to interact with others, to stay connected, and to share pictures, videos, or personal information^5,6^. The repetitive use of social media via smartphones and the reinforcement of reward expectations, can lead to habitual usage and impulsive responses to social media cues^7,8^, and also to the emergence of addiction-like symptoms in cases of loss of control and repeated use, despite negative consequences^8^.

A growing number of studies support the view that the uncontrolled use of the abovementioned online -communication applications can be a type of disorder. Yet, social-networks-use disorder (also termed Internet-communication disorder or social-networking-sites addiction), is not classified within respective classification manuals. In the 11^th^ revision of the *International Classification of Diseases (ICD-11)*^9^, only gambling disorder and gaming disorder are included as specific forms of disorders due to addictive behaviors. Both can be further subcategorized as occurring predominantly offline or online. The definitions of gambling disorder and gaming disorder include symptoms similar to those of disorders due to substance-use, i.e. impaired control over the behavior, increasing priority given to the behavior by neglecting other interests or activities, and continuation of the behavior despite negative consequences^9^. Even though social-networks-use disorder has not yet been officially classified, researchers already outline that this addiction-like behavior may be comparable to other behavioral addictions or substance-use disorders, at least along several dimensions^10–12^.

From a neuropsychological perspective, addictive behaviors (out of control behaviors that produce typical addiction symptoms) are assumed to result from an imbalance between two interacting neural systems. More specifically, it is assumed that a hyperactivity of an impulsive system, which enables quick and emotional responses towards immediately gratifying options, undermines cognitive control processes of a reflective system resulting in addictive behaviors; e.g.,^13–15^. The reflective system is associated with prefrontal cortex operations, such as executive functions and inhibitory control, which enable the control of impulsive responses^13,16–19^. According to Goldstein and Volkow^14^, dysfunctions in respective brain regions contribute to impairments in response inhibition. Situational factors (e.g. the presence of specific cues) and individual predisposing factors (e.g. personality traits) are assumed to interactively influence the way impulsive and reflective processes influence the decision to perform (or not to perform) a specific behavior see also^20^.

In accordance with such dual-process approaches and previous theoretical models^21–23^, the I-PACE (Interaction of Person-Affect-Cognition-Execution) model by Brand, *et al*.^24^ builds on empirical findings from behavioral and neuroimaging studies and summarizes the process of the development and maintenance of addictive behaviors. According to this model, individual predisposing variables, including trait impulsivity, affect the subjective perception of situational cues. Situational factors (e.g. confrontation with addiction-related cues, stress, and personal conflicts) are assumed to interact with individual coping styles and cognitive biases, leading to affective and cognitive responses, e.g. cue-reactivity, craving, and attentional bias^24,25^. Furthermore, inhibitory control and general executive functions, which encompass several cognitive processes such as retrieval and integration of information, cognitive flexibility, planning, monitoring, updating, strategy evaluation and application, as well as attention and inhibitory control^20,26,27^, buffer against addictive behavior enactment and the development of addiction symptoms. Reductions in general executive functions and inhibitory control intensify a specific behavior, e.g. the use of certain online applications, in a seemingly automatic way due to the anticipated experience of gratification and/or compensation. Brand, *et al*.^24^, therefore, positions general inhibitory control as a moderator in early stages of the addiction process, while in later stages, the so called “stimuli-specific reductions in inhibitory control” contribute to the maintenance of the respective addictive behavior. Such general and domain-specific cognitive control abilities can help social media users overcome tempting behaviors that are cued by the environment^28,29^. When such executive function and inhibition abilities are weak, conditioned learning and reinforcement mechanisms, such as stabilization and intensification, are augmented. This cycle could, over time, result in a loss of control over the behavior and thus in a continuous use of a specific online applications, despite negative consequences in everyday life.

The special relevance of impulsivity and general executive functions is highlighted by empirical findings on gambling disorder, gaming disorder, and other addictive behaviors related to application-specific Internet-use disorders. High impulsivity has been consistently associated with symptoms of specific Internet-use disorders e.g.^30–35^. For example, attentional impulsivity was shown to correlate with symptoms of gaming disorder^36^ and online pornography-use disorder^37^. General executive functions were found be reduced in individuals with gambling or gaming disorder^38–42^. Furthermore, a recent meta-analysis by Ioannidis, *et al*.^43^, demonstrated cognitive reductions (e.g., attentional and motor inhibition, decision making, working memory) in individuals with unspecified Internet-use disorder.

Specific inhibitory control has been relatively under-studied in the context of specific Internet-use disorders. Results of empirical studies investigating inhibitory control effects on addiction-related cues demonstrate inhibition deficits to be associated with gaming disorder and problematic online pornography use e.g.,^37,39,44^, but not with social-networks use disorder^45^. Possible interactions between impulsivity, general executive functions, and specific inhibitory control, as emphasized by theoretical models^21,24,25^, have been scarcely investigated in the context of specific Internet-use disorders. In one of the first studies addressing this interplay, Antons and Brand^37^ investigated impulsivity, craving, and specific inhibitory control in online pornography-users by using a Stop-Signal Task with pornographic cues. The authors concluded that the interactions between the mentioned factors contribute to problematic online behavior, and that this interplay should be further examined with other online behaviors and in other online contexts^37^.

In the context of social-networks use, only a few studies examined the effects of impulsivity and cognitive functions, albeit independently, on problematic use. They showed, for example, that impulsivity can be a risk factor for the development and maintenance of a social-networks-use disorder^46–48^; and that impairments in attentional control and inhibition abilities can be associated with problematic social media use behaviors^49–51^. Empirical studies on specific inhibitory control in social-networks-use disorder have also been rare. For example, Chung, *et al*.^52^ reported no significant associations between general inhibitory control assessed by a Go-NoGo paradigm (without addiction-related cues) and the symptom severity of a social-networks-use disorder. Moreover, Gao, *et al*.^53^ used a Go-NoGo paradigm with addiction-related cues in the context of social-networks-use disorder to show that individuals presenting social-networks-use disorder and healthy controls did not differ in Go-NoGo performance on a behavioral level. Lastly, brain regions involved in general and domain-specific self-control functions were not associated with social-networks-use disorder symptom severity^45,54–56^.

Possible interactions between impulsivity, general executive functions, and specific inhibitory control, indicating the interplay of impulsive and reflective processes have not yet been investigated experimentally in the context of social-networks-use disorder. Nevertheless, providing experimental data supporting the aforementioned interactions would have important implications for the current view of social-networks-use disorder. It can potentially more clearly indicating parallels to other addictive behaviors in terms of underlying psychological mechanisms. Looking at the interplay between such factors can also explain inconsistent and non-significant prior findings. For instance, it is possible that domain-specific inhibition was not significant in prior research because it is relevant only for people high in impulsivity trait. Overall, theoretical models and empirical findings allude to the idea that impulsivity, general executive functions, and specific inhibitory control can interact to influence the development and maintenance of addictive behaviors; and might therefore be involved in the problematic use of social networking sites. This study, therefore, addresses this gap and examines such interactions.

**Figure 1:**
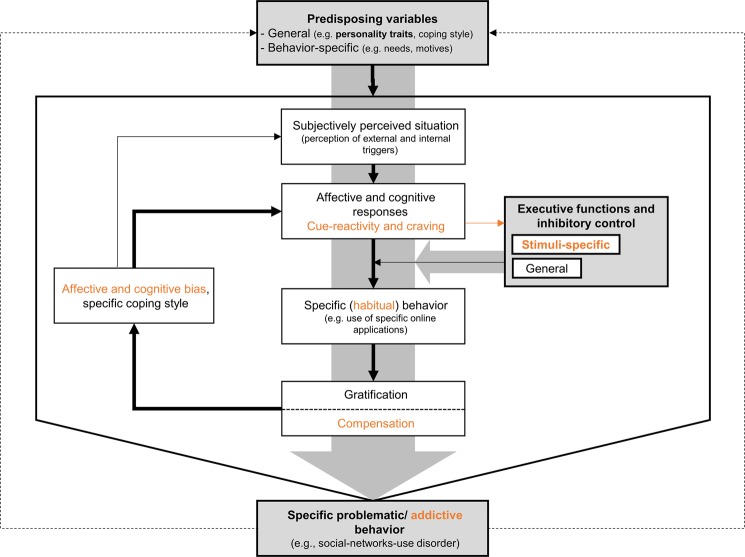
Illustration of the I-PACE model for specific Internet-use disorders modified from Brand, *et al*.^24^. The bold boxes and arrows highlighted in grey color indicate the components examined in the current study. Components in orange color represent those assumed for later stages of the addiction process^24^.

Specifically, the current study aims at investigating the roles of attentional impulsivity, reduced general executive functions and diminished specific inhibitory control in explaining social-networks-use disorder symptom severity. Based on the I-PACE model, we hypothesize that (attentional) trait impulsivity interacts with general executive functions and specific inhibitory control in the prediction of social-networks-use disorder symptoms (see Fig. 1 for an illustration of the hypothesized model). We expect that attentional impulsivity translates into social-networks-use disorder symptoms, especially when specific inhibitory control is diminished and general executive functions are impaired. In order to examine addiction-specific inhibitory control in the context of social-networks-use disorder, we implemented an auditory Go-NoGo task including ringtones of popular online-communication applications as social-networks-specific cues. These ringtones are considered to have a demanding character in everyday life and to be mainly associated with online-mediated social interactions.

## Results

The sum score of the sIAT-SNS indicating social-networks-use disorder symptoms mean score of *M* = 23.82, *SD* = 7.02 (range = 13 to 46). Following the main aim of our study, we examined the effect of measures of reduced general executive functions (Stroop, TMT-B, MCST) and reduced specific inhibitory control (auditory Go-NoGo paradigm) on the symptom severity of social-networks-use disorder (sIAT-SNS), both separately, as well as in interaction with trait impulsivity (BIS-15). Therefore, in a first step, we calculated bivariate correlations between the mentioned measures. In a second step, we analyzed how impulsivity interacts with general executive functions and specific inhibitory control in the prediction of social-networks-use-disorder symptoms. Therefore, we performed moderated regression analyses (see below) with three steps using mean-centered variables^57^.

### Correlation analysis

Correlation analysis indicated that the level of symptoms of social-networks-use disorder (s-IAT-SNS) is significantly positively associated with the attentional subscale of the BIS-15, but not with any other measure (see Table 1).

**Table 1:** Pearson correlations between measures of social-networks-use-disorder symptoms (sIAT-SNS), impulsivity, general executive functions, and specific inhibitory control.

	*M (SD)*	sIAT-SNS	(1)	(2)	(3)	(4)	(5)	(6)	(7)	(8)	(9)
(1) BIS-15 non-planning	2.17 (0.61)	0.179	1								
(2) BIS-15 motor	2.40 (0.51)	0.162	0.462**	1							
(3) BIS-15 attentional	1.98 (0.49)	0.361**	0.256**	0.366**	1						
(4) Stroop 3 (time in sec)	65.12 (12.34)	0.041	−0.104	0.041	0.001	1					
(5) TMT-B (time in sec)	51.38 (17.81)	0.010	0.016	0.004	−0.079	0.334**	1				
(6) MCST pers. mistakes	2.78 (3.00)	0.052	−0.070	−0.038	−0.149	0.185	0.225*	1			
(7) false Go SNS	1.02 (1.55)	0.101	−0.039	0.161	0.098	0.482**	0.379**	0.205*	1		
(8) false Go neutral	2.13 (1.95)	0.050	−0.158	−0.025	−0.077	0.317**	0.459**	0.211*	0.561**	1	
(9) false NoGo SNS	1.70 (2.24)	0.074	0.010	0.226*	0.126	0.269**	0.170	0.090	0.503**	0.291**	1
(10) false NoGo neutral	5.69 (4.01)	−0.051	−0.010	0.036	0.044	0.210*	0.266**	0.109	0.315**	0.479**	0.494**

*Notes*. BIS = Barratt Impulsiveness Scale; TMT-B = Trail Making Test, part B; MCST = Modified Card Sorting Test; SNS = Social networking sites *p ≤ 0.050, **p ≤ 0.010.

### Prediction of the symptom severity of social-networks-use disorder

We next analyzed possible interaction effects using multiple hierarchical regression analyses^57^. Post-hoc simple slope analyses were performed to illustrate significant interaction effects^58^. In each of the analyses, sIAT-SNS was the dependent variable. Previous empirical studies emphasize the special relevance of attentional impulsivity. Therefore, we used attentional impulsivity (BIS-15 attentional) as first predictor. Each analysis included another measure of general executive functions or specific inhibitory control as the second predictor, resulting in seven different analyses. The respective interaction terms were calculated using mean-centered values of the first and second predictors^57^.

#### Interactions between impulsivity and general executive functions

**Figure 2:**
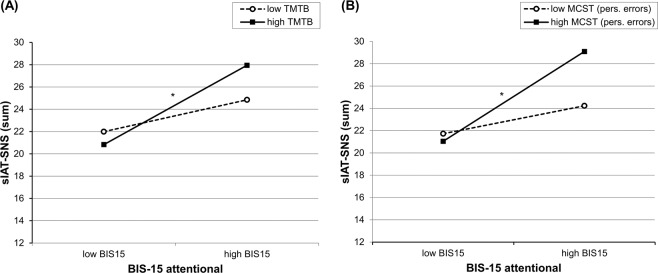
Simple slopes for interaction effects of attentional impulsivity with two measures of general executive functions: (**A**) Trail Making Test (TMT-B) and (**B**) Modified Card Sorting Test (MCST) perseverative errors. Variables were grouped one standard deviation above (“high”) and below (“low”) the mean values^58^. High values in TMT-B and MCST respectively represent low executive functions. *Slope is significant at *p* < 0.05.

As measures of general executive functions, the performance scores in the Stroop test (seconds needed in part 3), the TMT-B (seconds needed), and the MCST (perseverative errors) were each included within a separate analysis. The results revealed that attentional impulsivity, in the first steps, significantly explained 13% of the variance sIAT-SNS, *F*(1,111) = 16.44, *p* < 0.001. The addition of any of the general executive function measures, in the second steps, did not lead to further variance explanation (all Δ*R*² ≤ 0.002, all *p’*s > 0.05). However, the interactions between BIS-15 attentional × TMT-B (Δ*R*² = 0.034, Δ*F* = 4.44, *p* = 0.037) and BIS-15 attentional × MCST (Δ*R*² = 0.032, Δ*F* = 4.17, *p* = 0.044) were significant in explaining additional ~3% of the variance in sIAT-SNS. Both overall models were significant in explaining variance of the sIAT-SNS (TMT-B as moderator: *R²* = 0.166, *F*(3,111) = 7.16, *p* < 0.001, MCST as moderator: *R²* = 0.173, *F*(3,111) = 7.55, *p* < 0.001). Looking at the simple slopes revealed a similar picture for both interactions: sIAT-SNS was the highest in individuals with high attentional impulsivity (indicated by high BIS-15 attentional) and low general executive functions (indicated by high time needed in the TMT-B, *t* = 4.64, *p* ≤ 0.001, or high errors in the MCST, *t* = 4.41, *p* ≤ 0.001). Individuals with high attentional impulsivity had significantly higher levels of symptoms of social-networks-use disorder only if, additionally, general executive functions were low. The significant interaction effects are illustrated in Fig. 2 (A: interaction with TMT-B; B: interaction with MCST). The interaction with the Stroop variable was not significant (see Table 2 for the statistics of the coefficients).

**Table 2:** Statistics of the coefficients of the moderated regression analyses predicting symptom severity of social-networks-use disorder.

		*B*	*SE*	β	*T*
General executive functions	BIS-15: attentional	5.13	1.28	0.335	4.02**
	TMT-B (time in sec)	0.03	0.04	0.070	0.78
	Interaction (BIS-15 × TMT-B)	0.12	0.06	0.188	2.11*
	BIS-15: attentional	5.43	1.28	0.376	4.25**
	MCST (perseverative errors)	0.35	0.21	0.149	1.64
	Interaction (BIS-15 × MCST)	0.94	0.46	0.183	2.04*
Specific inhibitory control	BIS-15: attentional	4.83	1.28	0.335	3.78**
	Go-NoGo: false Go SNS (sum)	0.20	0.40	0.043	0.49
	Interaction (BIS-15 × Go-NoGo)	1.57	0.74	0.188	2.11*

*Notes*. BIS = Barratt Impulsiveness Scale; TMT-B = Trail Making Test, part B; MCST = Modified Card Sorting Test; SNS = Social networking sites; *p ≤ 0.050, **p ≤ 0.010.

#### Interactions between impulsivity and specific inhibitory control

The variables of the new auditory Go-NoGo paradigm modified for SNS served as measures of specific inhibitory control. Following the above-described procedure, attentional impulsivity (BIS-15 attentional) was included as the first predictor and, in separate analyses, each of the Go-NoGo scores (false-Go-SNS, false-Go-neutral, false-NoGo-SNS, false-NoGo-neutral) were included as the second predictor, respectively. The dependent variable was again sIAT-SNS, capturing symptom severity of social-networks-use disorder.

**Figure 3:**
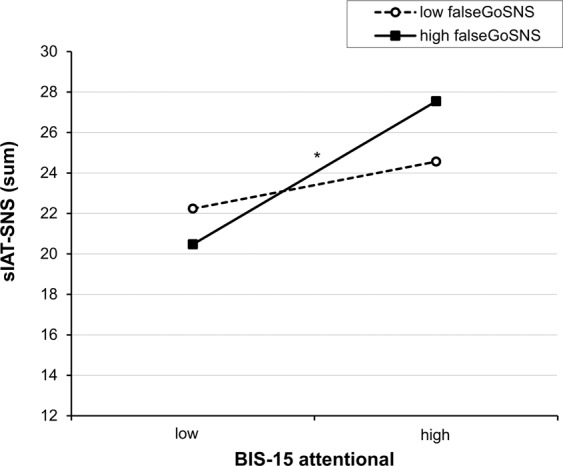
Simple slopes for the interaction effect of attentional impulsivity with performance in the auditory Go-NoGo task. Variables were grouped one standard deviation above (“high”) and below (“low”) the mean values^58^. High values in the Go-NoGo task represent weak performance. *Slope is significant at *p* < 0.05.

The results showed that, beyond the already reported effect of attentional impulsivity, none of the Go-NoGo variables had an additional effect (all Δ*R*² < 0.007, all *p’*s > 0.05). However, there was a significant interaction between false-Go-SNS with BIS-15 attentional on sIAT-SNS, Δ*R*² = 0.034, Δ*F*(1,108) = 4.47, *p* = 0.037. The overall model was significant in explaining 16.9% of the variance in sIAT-SNS, *F*(3,111) = 7.31, *p* < 0.001. The simple slopes analysis illustrated that individuals with high attentional impulsivity (BIS-15 attentional) had a significantly higher sIAT-SNS scores than those with low attentional impulsivity but particularly if accompanied by high false-Go-SNS, *t* = 4.45, *p* < 0.001, rather than low false-Go-SNS, *t* = 1.32, *p* = 0.189. Accordingly, social-networks-use-disorder symptoms are the highest in individuals with weaknesses in both attentional impulsivity and Go-reactions on SNS cues. Figure 3 depicts the significant interaction effect (see Table 2 for coefficients’ statistics).

In contrast, the interaction between BIS-15 attentional and false-NoGo-SNS had no additional effect, Δ*R*² = 0.001, Δ*F*(1,108) = 0.16, *p*> 0.05. The interactions with false reactions on neutral trials also did not contribute to additional variance explanation, neither for Go trials (false-Go-neutral), Δ*R*² = 0.012, Δ*F*(1,108) = 1.51, *p* > 0.05, nor for NoGo trials (false-NoGo-neutral), Δ*R*² < 0.001, Δ*F*(1,108) = 0.05, *p* > 0.05.

## Discussion

The current study investigated the importance of considering impulsivity, general executive functions, and specific inhibitory control in tandem, for understanding and explaining the development and maintenance of a social-networks-use disorder. The results suggest that attentional impulsivity is related to a higher symptom severity of social-networks-use disorder. At the same time, we found no direct associations of general executive functions and specific inhibitory control with symptom severity of social-networks-use disorder. However, there were several interaction effects emphasizing that individuals with higher attentional impulsivity are especially predisposed to develop stronger symptoms of a social-networks-use disorder if they additionally show reductions in general executive functions and social-networks-specific inhibitory control.

The results are consistent with former research illustrating associations between impulsivity and symptoms of social-networks-use disorder^46–48^ and of other specific Internet-use disorders e.g.,^33,36,59^. For example, the reward system in the brain (amygdala-striatal system), which creates states of motivation for impulsive behaviors, and that has been implicated across various impulsive behaviors, has been found to be hyper-active^45^, structurally pruned and more efficient^54,55^ in people with stronger social-networks-use disorder symptoms. Indeed, in their review, Mitchell and Potenza^60^ summarized the critical role of impulsivity by highlighting that personality traits associated with more impulsive behavior are related to several forms of substance-use disorders. They further mention interactions between different facets of impulsive behavior (i.e. interactions between impulsivity and sensation-seeking) to be associated with substance use. In that sense, our findings are in line with prior research that highlights the importance of impulsivity in promoting risky social-networks-use behaviors^48^ and addictive social-networks use^61^. These effects are rooted in part in dual-system theories^62^ as also applied to social networking sites^23^; according to which impulsivity is a manifestation of the hyperactivity of the brain system that govern reward expectation and processing (system 1)^63^.

The I-PACE model by Brand, *et al*.^24^ extends and contextualizes such dual system models of addictive behaviors to the case of Internet applications. Based on this model, we hypothesized that the interactions between personality traits and general executive functions, as well as with specific inhibitory control can result in a diminished control over the behavior. Our results confirmed this hypothesis. If impulsivity appears together with either reduced general executive functions or impaired specific inhibitory control of social-networks related cues, then individuals have higher symptom severity of social-networks-use disorder compared to those without impairments in general executive functions or specific inhibitory control. Antons and Brand^37^ showed similar interaction effects between impulsivity and specific inhibitory control for male participants with problematic online pornography use. Results of a later study^64^ showed that the attentional domain of impulsivity differentiates recreational from unregulated pornography use. Our current results indicate that attentional impulsivity is an especially relevant facet in the context of social-networks-use disorder as well. This points to a similar role of impulsivity across various problematic online behaviors, and to the generalizability of the core dual-system and I-PACE ideas across various problematic online behaviors. Together, the findings emphasize that the presentation of specific Internet-related stimuli affects attentional processes, which could result in diminished control over the use of Internet/social-networks, if accompanied by high attentional impulsivity as a predisposing variable. This view has also been supported from a neuroscientific standpoint, by studies showing that activation and morphology of centers of impulsivity and diminished ability to overcome impulsions are associated with social-networks-use disorder symptom severity^45,56^.

The current findings illustrate that especially those variables that are associated with deficits in attention and information processing interact with higher attentional impulsivity, which ultimately results in higher levels of social-networks-use disorder symptoms. The special relevance of attention-related facets of inhibitory control is indicated by the fact that interactions only occurred with false “Go” (and not “NoGo”) reactions on online-communication-related cues. Interestingly, general executive functions assessed by the MCST (such as visual attention, switching, rule detection, feedback processing, and cognitive flexibility) show similar interaction patterns with attentional impulsivity, which supports theoretical assumptions on addictive Internet use^25,65^. However, there was no significant interaction between impulsivity and general executive functions measured by the Stroop test, which is often used as a measure of general inhibition capability. This may indicate that “higher-level” executive functions (as mainly measured by the MCST), such as updating and set-shifting, and “lower-level” executive functions (as mainly measured by the Stroop test), such as inhibition ability e.g.,^27,66,67^, are differentially involved in mediating addictive behaviors online. We call for future research to delve deeper into such distinctions.

The reported interactions between the different components further fit dual-process perspectives, which suggest that addictive behaviors result from an imbalance between impulsive and reflective processes^13,68,69^. A dominance of the impulsive system is assumed to induce approach tendencies towards potentially gratifying options while neglecting long-term risks, which may result in risky behavior such as drug consumption e.g.,^68,70^. These approach tendencies could be triggered and/or reinforced by facing specific cues related to the expected gratification. In the context of smartphone use, a ringtone or auditory signal associated with an online-communication application can be one such cue. Smartphones, and especially certain cues associated with new incoming information or messages, have an explicit demanding character and are able to induce craving to use online-communication applications^71^. Auditory smartphone notifications were shown to attract attention and affect task performance, especially in individuals with a tendency towards an excessive use^72^. Cognitive control processes are necessary in order to inhibit or ignore impulsive responses on demanding cues in situations, when responding to the auditory signals is not appropriate. The current findings support the assumption that individuals with reductions in both attention and general executive functions are especially prone to develop stronger social-networks-use disorder symptoms when they are impulsive. The observed interaction effects are in line with prior research on impulsive alcohol consumption^73^ and social media use^28,29^; such studies showed that cognitive-emotional preoccupation with the behavior (akin to and/or associated with impulsivity) increases problematic behaviors, and that this effect is augmented by weak cognitive behavioral control (akin to and/or associated with executive functions). We hence call for future research to integrate these perspectives and provide a more fine-grained explanation of the interactions we observed here.

Following the I-PACE model^24^, facing a demanding cue could be described as an additional reinforcement mechanism, resulting in that exercising control over the behavior becomes increasingly difficult over time. It is possible that cue activation strength alone can lead to social-networks-use disorder symptoms, even if executive control is intact; the hyper-sensitization of the cue processing system in the brain can create a strong motivation state that even a healthy prefrontal cortex may not be able to handle^45^. Further research should address the demanding character of smartphones and online-communication applications as well as their effect on the individual’s attention more systematically. The investigation of possible long-term effects, such as attentional deficits, when cognitive control is additionally reduced or distracted is important for better understanding the impact of the use of social media on human (social) behavior. For example, attention deficits have been linked to risky online behavior, such as checking social media while driving; and such risky behaviors have immense adverse societal implications^48^. Our findings allude to the idea that, besides the investigation of mechanisms for the development and maintenance of an addictive behavior, examining the interplay of mobile phone cues (specially auditory ones), impulsivity, general executive function and domain specific inhibition abilities may be relevant for the investigation of a broader set of emotional-cognitive deficits and their associated behaviors.

The use of auditory tones as cues is also noteworthy. First, it extends prior insights on dual-system deficits that underlie automatic responses and addictive behaviors; such studies typically used visual cues^45^ and we show here that auditory cues on social media can also trigger automatic responses and addictive behaviors. Furthermore, our findings can inform the debate about the concept of “smartphone addiction” or disordered smartphone use. While some argue that people can develop addiction-like symptoms in relation to smartphone use^74–77^, others suggest that people develop such symptoms in relation to specific applications, that are mediated, in part (not always) via the smartphone^78^ and that such symptoms rarely meet addiction criteria^79^. While we do not resolve this debate here, we show that common features of smartphone, such as auditory cues, can underlie the development and maintenance of strong motivation states that may manifest in addiction-like symptoms in relation to social media use via the smartphone. That is, smartphones can serve as efficient channels that afford and facilitate impulsive behaviors on communication applications by affording the provision of auditory cues. Future research can examine whether and how other smartphone features, such as “waking up” when a new message arrives, contribute to addiction symptom formation and maintenance, and whether these effects are unique to smartphones, or can accrue also in other technologies that mediate communication applications (e.g., desktops, tablets).

Importantly, the results of the current study revealed no direct relationships between reduced general executive functions, specific inhibitory control, and social-networks-use disorder symptoms. This is in contrast to studies reporting diminished inhibitory control (Stroop and Go-NoGo tasks) in addictive behaviors^60,80^. The recent meta-analysis by Ioannidis, *et al*.^43^ illustrates cognitive reductions in individuals showing addictive unspecified Internet use. However, in contrast to the current study that focused on relatively normal users (i.e., who do not typically meet clinical classification criteria), the meta-analysis findings focused on differences between addicted individuals (those who meet common addiction criteria) and healthy controls. Impairments in general executive functions and especially specific inhibitory control are assumed to occur in later stages of the addiction process^24^, which may explain the missing direct associations in the current sample including individuals who are at most in an early stage or at risk of developing social-networks-use disorder. This is in line with neuroscientific evidence that disordered social media use symptoms in non-clinical samples are typically not associated with prefrontal brain impairments, which would manifest in reduced executive function and inhibition^45,54–56,81^. Future research should examine when and how prefrontal brain impairment, reduced executive function, and inhibition abilities contribute to the transition of impulsive non-clinical populations into significant impairments to normal functioning that can be classified as social-networks-use disorder cases.

The findings have implications for prevention and treatment programs. It seems that individuals with high attentional impulsivity are at a higher risk for developing social-networks-use disorder symptoms. This is consistent with prior research demonstrating attention-deficit/hyperactivity disorder as a comorbidity of social-networks-use disorder e.g.,^82^. Given our findings (the moderation effects), the strengthening of general executive functions could be an important preventive mechanism for individuals suffering from attention-deficit/hyperactivity disorder or general higher attentional impulsivity. Furthermore, improving specific inhibitory control when confronted with certain cues might be a promising avenue for treatment and for maintaining abstinence^60^. In addition, because auditory cues can create strong motivation states, removing such cues (e.g., by silencing the phone) can help people prevent and reduce impulsive use of communication applications. Additionally, interactions between impulsivity and general executive functions as well as specific inhibitory control should be kept in mind in the context of clinical treatment^83^. Neuropsychological training could support the reflective system and respective cognitive resources in suppressing impulsive responses. Future research should also look into stress reduction techniques (e.g. meditation) and executive function enhancement (e.g., mindfulness) as a means to improve people’s inhibition abilities in relation to social-networks use.

Several limitations of this study are noteworthy. First, we cannot infer causal relations from the results. Like in the case of substance-use disorders^60^, it can be assumed that impulsive tendencies are associated with a specific neurobiological constitution, which serves as a predisposing factor increasing the susceptibility for developing an addictive behavior (social-networks-use disorder in our case), but that this association might be altered with progression in the addiction process. Thus, future research can employ longitudinal designs to examine how the associations we observed here change overtime as the disorder symptoms develop. Second, the auditory Go-NoGo paradigm with addiction-related cues is a newly developed instrument that needs further validation. Future studies could use the paradigm for assessing specific inhibitory control in other behavioral addictions, e.g., the effect of the auditory signal of a slot machine in individuals with gambling disorder. However, the current results illustrate correlations between the auditory Go-NoGo paradigm and the instruments for measuring general executive functions comparable to the study by Wegmann, *et al*.^84^, which provides initial evidence regarding the validity and reliability of the paradigm (see Supplementary Information). A further limitation is the low reliability of the subscale “attentional impulsivity” of the Barratt Impulsiveness Scale^85^. Given that the subscale has five items only, which represent different facets of attentional impulsivity, the internal consistency may not be the best measure for reliability. Since the internal consistency of the subscale in our sample is comparable to previous studies investigating the validity and reliability of the questionnaire e.g.,^86,87^, we think that this low reliability is not specific for the current sample and does not indicate a sample-specific systematic measurement error. However, the relatively low internal consistency of this subscale should be taken into account when generalizing the results of our study. Although the questionnaire has been used globally in hundreds of studies, it may be worth considering the psychometric properties of the scale in future studies including a large and representative sample.

To conclude, the current findings add to studies emphasizing the relevance of facets of state and trait impulsivity for the development of addictive behaviors (online and offline) by showing respective associations in the context of social-networks-use disorder. They further extend the range of cues that motivate social media use from visual to auditory. However, even if the results emphasize the relevance of investigating the interaction between impulsivity, and general executive functions as well as specific inhibitory control, we have to keep in mind that the effect size of the interaction effect is relatively low, explaining approximately 3% of the variance in a non-clinical sample. We argue, however, that this effect is meaningful, given that typically effects in a clinical sample with a larger variance of symptom severity are stronger compared to effects found in non-clinical samples. Theoretical assumptions about the interactions between personality traits, general executive functions, and specific inhibitory control, and their effects on tendencies towards specific Internet-use disorders are supported in the case of use of online-communication applications.

## Methods

### Participants

In the current study, we investigated 112 participants (out of which 63 were female) aged between 17 and 53 years old (*M* = 22.76, *SD* = 7.11). Most of the participants were apprentices or students (83.9%), 49.2% lived in a romantic relationship, and 6.3% had children. The majority (72.3%) reported to have a general qualification for university entrance, and the rest either graduated university (10.8%) or post-high-school institution (16.1%), or provided no information (0.9%). The majority (93 participants) scored non-problematic, 15 participants scored problematic, and 4 participants reached the cutoff for a pathological use on the social-networks-use disorder scale. This non-clinical, convenient sample was recruited by mailing lists and contact lists of the University of Duisburg-Essen. The study was conducted in a laboratory, individual stetting. The procedure was approved by the local ethics committee of the division of Computer Science and Applied Cognitive Sciences at the Faculty of Engineering of the University of Duisburg-Essen in accordance with the Declaration of Helsinki. All participants gave written informed consent by signing a declaration of consent. Germany allows self-reliant participation from the age of 14 onwards in case the content is not morally harmful to youth, which has been approved by the local ethics committee as well. In Germany, university students from the age of 16 are allowed to participate in studies without the confirmation by the parents, since for participation they receive credits for university courses. The current study included participants at the age of 17 and older.

A power analysis using G*Power (version 3.1.9.2) revealed a power of 0.93 given a medium effect size for multiple regression analyses (f² = 0.15; based on Cohen^88^) and a sample size of N = 112. The respective estimation revealed that a sample size of at least N = 77 would have been necessary to detect a medium-sized effect size with a power of 0.80^88^.

### Instruments

#### Symptom severity of social-networks-use disorder

The severity of symptoms of social-networks-use disorder was measured with the short Internet Addiction Test modified for social-networking sites (sIAT-SNS)^89^. The questionnaire measures the symptom severity and subjective impairments in everyday life due to the use of social media and online-communication applications without any time specification. In the instructions, a definition of social-media use is given including definitions of active and passive usage. Participants are asked to rate 12 items on a five-point Likert scale ranging from 1 (=never) to 5 (=very often). The sum score can range from 12 to 60, whereby scores > 30 indicate problematic and scores > 37 indicate pathological use^90^. We use these classifications for descriptive purposes, which are in line with recent studies^89,90^, and not in the analysis, given the embryonic stage of classification systems and that there is still no agreed upon cut-offs. Cronbach’s α was 0.859.

#### Trait impulsivity

Trait impulsivity was assessed with the German short version of the Barratt Impulsiveness Scale named BIS-15^85^. The BIS-15 consists of 15 items (three subscales with five items each) rated on a four-point Likert scale (1 = rarely/never to 4 = almost always/always). The sum scores of the three subscales serve as measures of non-planning (e.g., “I plan tasks carefully”; Cronbach’s α = 0.813), motor (e.g., “I act on impulse”; Cronbach’s α = 0.639), and attentional impulsivity (e.g., “I don’t pay attention”; Cronbach’s α = 0.598), respectively.

#### General executive functions

Three instruments for measuring general executive function were used: (1) the Stroop Color-Word-Interference Test^91^ measuring information processing and resistance to cognitive interference, (2) part A and B of the Trail Making Test^92,93^ measuring visual attention and switching, and (3) a computerized version of Nelson’s^94^ Modified Card Sorting Test (MCST) as a measure of cognitive functions such as rule detection, feedback processing, and cognitive flexibility. In the MCST, errors due to perseveration in the previous rule despite explicit information that the rule has changed are summed as perseverative errors. Other incorrect sorting are summed as non-perseverative errors. We used these different measures, since each of them is assumed to cover slightly different aspects of executive functions see also^95,96^.

#### Specific inhibitory control

**Figure 4:**
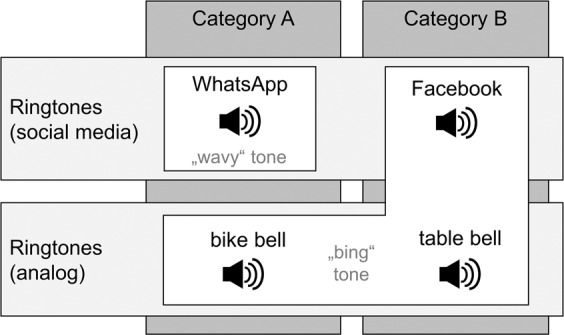
Visualization of the cues used in the auditory Go-NoGo task including social-networks-related ringtones. Each category contains two stimuli with different attributes (social media vs. analog and “wavy” vs. “bing”). The presentation of the stimuli were pseudorandomized see also^84^.

An auditory Go-NoGo paradigm modified with social-networks-related cues was used to capture specific inhibitory control. The modified version was based on the auditory Go-NoGo paradigm by Wegmann, *et al*.^84^ and the concepts of the visual version by Verdejo-García and Pérez-García^97^. As auditory cues, we used four different kinds of ringtones of comparable length. The cues included digital (social-networks related) and analog (neutral) ringtones, which in part overlap semantically in a systematic manner (see Fig. 4). The digital ringtones included two social-networks-related cues: the standard tones for incoming messages on Facebook and the instant messenger service WhatsApp (owned by Facebook). The analog ringtones included two neutral cues, not related to online-communication applications: the rings of a table bell and a bike bell. The analog ringtones were chosen carefully in order to additionally ensure similarities with one of the social-networks-related tones following the taxonomy of Go-NoGo tasks introduced by Verdejo-García and Pérez-García^97^ and Wegmann, *et al*.^84^.

The instruction phase was as follows: First, participants were instructed to react (Go trials) by pressing a specific key or not-react (NoGo trials) to different auditory cues. Second, each of the four different cues was introduced along with a pictorial presentation of the source of the respective tone (e.g., the Facebook tone along with the Facebook logo). This introductory step aimed to ensure that the cues are recognized correctly as digital or analog ringtones, respectively. In the actual Go-NoGo task, the cues were presented only auditory. Third, participants performed 20 practice trials (10 Go trials and 10 NoGo trials) in order to become familiar with the task procedure.

The actual task consisted of four rounds of 20 trials each, resulting in a total of 80 trails. In each round, every cue was presented five times without direct repetition. Before each round, participants were instructed on which two tones they should react by button press (Go trials), and on which two tones they should not react (NoGo trials). Go trials occurred no more than twice in succession. After each round, the associations between the Go and NoGo tones reversed. Performance was measured as the sum of false reactions in Go and independently, in NoGo trials. Both sum scores were additionally separated by type of cue, namely digital and analog ringtones (neutral). False reactions in Go trials served as an indicator of deficits in attention and information processing, and false reactions in NoGo trials indicated reduced inhibitory control^84^. Presentation ® software was used to perform the task (Version 16.5, www.neurobs.com).

The different reaction patterns and correlations with other tests are similar to those reported for the original auditory Go-NoGo task (see Supplementary Table S1 for additional descriptive statistics on Go-NoGo task performance and Supplementary Table S2 for correlations between the Go-NoGo paradigm and the three measurements for general executive functions).

### Statistical analyses

Statistical analyses were carried out with SPSS 26.0 (IBM Statistics). Pearson correlation coefficients were calculated to test the bivariate correlations between two variables, with r ≥ 0.01 indicating a small, r ≥ 0.03 indicating a medium, and r ≥ 0.05 indicating a high effect size^88^. To test the effects of different predictors and their interactions on the dependent variable, multiple hierarchical moderated regression analyses were calculated. All predictors were centralized. Significant interaction effect were further analyzed by using simple slope analyses.

## Supplementary information

Supplementary Information.

## Data Availability

Data will be made available for research use purposes, upon request.
