# Supplementary material for: Interactions of impulsivity, general executive functions, and specific inhibitory control explain symptoms of social-networks-use disorder: An experimental study
Source: Sci Rep. 2020 Mar 2;10:3866. doi: 10.1038/s41598-020-60819-4 (PMC7052241; doi:10.1038/s41598-020-60819-4)
Supplement: Supplementary file 1 — Supplementary Information. [file 41598_2020_60819_MOESM1_ESM.pdf]

# Interactions of impulsivity, general executive functions, and specific inhibitory control explain symptoms of social-networks-use disorder: An experimental study

Elisa Wegmann[1], Silke M. Müller[1,2], Ofir Turel[3,4], & Matthias Brand[1,2]

[1] Department of General Psychology: Cognition and Center for Behavioral Addiction Research (CeBAR), University of Duisburg-Essen, Duisburg, Germany

[2] Erwin L. Hahn Institute for Magnetic Resonance Imaging, Essen, Germany

[3] Information Systems and Decision Sciences, California State University, Fullerton, CA, USA

[4] Brain and Creativity Institute, University of Southern California, Los Angeles, USA

**Corresponding author:**

Matthias Brand (matthias.brand@uni-due.de)

University of Duisburg-Essen

General Psychology: Cognition and Center for Behavioral Addiction Research (CeBAR)

Forsthausweg 2

47057 Duisburg

Germany

Phone: ++49-203-3792541

Fax: ++49-203-3791846

**Supplementary Table S1**: *Descriptive statistic of reactions in the auditory Go-NoGo paradigm.*

| | Range | *M* | *SD* |
|---|---|---|---|
| Number of False NoGo trials | | | |
| SNS 1 | 0-6 | 0.72 | 1.16 |
| SNS 2 | 0-7 | 0.97 | 1.50 |
| Neutral 1 | 0-9 | 3.08 | 2.35 |
| Neutral 2 | 0-8 | 2.61 | 2.12 |
| Sum | 0-27 | 7.38 | 5.47 |
| Number of False Go Trials | | | |
| SNS 1 | 0-4 | 0.46 | 0.78 |
| SNS 2 | 0-7 | 0.55 | 1.15 |
| Neutral 1 | 0-4 | 1.11 | 1.06 |
| Neutral 2 | 0-6 | 1.03 | 1.33 |
| Sum | 0-15 | 3.15 | 3.10 |

Regarding the modified stimuli, overall, participants made fewer false reactions on SNS stimuli than on neutral stimuli in both Go trials, $t = -7.03$, $p < .001$, and NoGo trials, $t = -12.10$, $p < .001$.

**Supplementary Table S2**: *Descriptive statistics of measures of impulsivity and general executive function and their correlations with auditory Go-NoGo task performance.*

| | | | SNS | | Neutral | | Total |
|---|---|---|---|---|---|---|---|
| | ***M*** | ***(SD)*** | **Go** | **NoGo** | **Go** | **NoGo** | **Sum** |
| BIS-15 non-planning | 2.17 | (1.55) | -.039 | .010 | -.158 | -.010 | -.051 |
| BIS-15 motor | 2.40 | (2.24) | .161 | .226* | -.025 | .036 | .113 |
| BIS-15 attentional | 1.98 | (1.95) | .098 | .126 | -.077 | .044 | .061 |
| Stroop 1 (time in sec) | 28.65 | (4.01) | .342** | .179 | .320** | .164 | .293** |
| Stroop 2 (time in sec) | 42.65 | (7.55) | .459** | .285** | .350** | .141 | .344** |
| Stroop 3 (time in sec) | 65.12 | (0.61) | .482** | .269** | .317** | .210* | .372** |
| TMT A (time in sec) | 24.93 | (0.51) | .095 | .043 | .249** | .120 | .160 |
| TMT B (time in sec) | 51.38 | (0.49) | .379** | .170 | .459** | .266** | .388** |
| MCST non-perseverative mistakes | 4.46 | (4.78) | .091 | .075 | .167 | .088 | .131 |
| MCST perseverative mistakes | 2.78 | (8.07) | .205* | .090 | .211* | .109 | .182 |

* $p \leq .050$

** $p \leq .010$
